# Pisa Syndrome in Parkinson's Disease: Electromyographic Aspects and Implications for Rehabilitation

**DOI:** 10.1155/2015/437190

**Published:** 2015-11-23

**Authors:** Giuseppe Frazzitta, Pietro Balbi, Francesco Gotti, Roberto Maestri, Annarita Sabetta, Luca Caremani, Laura Gobbi, Marina Capobianco, Rossana Bera, Nir Giladi, Davide Ferrazzoli

**Affiliations:** ^1^Department of Parkinson's Disease, Movement Disorders and Brain Injury Rehabilitation, “Moriggia-Pelascini” Hospital, Gravedona ed Uniti, 22015 Como, Italy; ^2^Department of Neurorehabilitation, “Salvatore Maugeri” Foundation, IRCCS, Scientific Institute of Pavia, via Boezio, 27100 Pavia, Italy; ^3^Department of Biomedical Engineering, S. Maugeri Foundation, IRCCS, Scientific Institute of Montescano, 27040 Pavia, Italy; ^4^Movement Disorders Unit, Neurological Institute, Tel Aviv Medical Centre, Sieratzki Chair of Neurology, Sackler School of Medicine, Sagol School for Neuroscience, Tel-Aviv University, 69978 Tel-Aviv, Israel

## Abstract

Pisa Syndrome (PS) is a real clinical enigma, and its management remains a challenge. In order to improve the knowledge about resting state and during maximal voluntary muscle contraction (MVMC) of the axial muscles, we described the electromyography results of paraspinal muscles, rectus abdominis, external oblique, and quadratus lumborum of both sides of 60 patients. Electromyography was assessed at rest, during MVMC while bending in the opposite direction of the PS and during MVMC while bending in the direction of the PS. The MVMC gave information about the interferential pattern (INT) or subinterferential pattern (sub-INT). We defined asymmetrical activation (AA) when a sub-INT was detected on the muscle on the side opposite to the PS bending and an INT of same muscle in the direction of PS bending. We observed significant AA during MVMC only in the external oblique muscles in 78% of the subjects. Our results of asymmetric ability to generate maximal voluntary force of the external oblique muscles support a central dissynchronisation of axial muscles as a significant contributor for the bending of the spine in erect position. These results could have important implication to physiotherapy and the use of botulinum toxin in the treatment of PS.

## 1. Introduction

Parkinson's disease (PD) is one of the most common neurodegenerative diseases, and abnormal trunk's postures represent an important source of disability for parkinsonian patients. Among them, the Pisa Syndrome (PS) is a real clinical enigma, and its management remains a challenge. It was first described as an acute axial dystonia related to the administration of neuroleptics [1]. It is clinically defined as a sustained lateral bending of the trunk (at least 10°), worsened by prolonged sitting position or walking and completely disappearing in lying position [2]. However, the lack of consistent diagnostic criteria led to significant differences in frequency reports (described in 2 to 90% of parkinsonian patients) and has prevented the research from progressing in its pathophysiological mechanism [3].

The discussion about the central or peripheral origin of PS is still active: some authors believe that the lateral flexion of the trunk in PD is an axial dystonia [4], while others suggest an abnormal proprioception of axial posture as the primary cause of PS [5]. Moreover, few studies suggested peripheral causes, in the form of paraspinal myopathy or skeletal and soft tissue changes, as the underlying pathophysiological mechanism leading to PS [6, 7].

Treatment of PS is still a challenge: there is no effective pharmacological therapy and deep brain stimulation of the subthalamic or the pedunculopontine nucleus, reported to have some benefit, is used as last resource [8–10]. Recently, botulinum toxin (BTX) injection of axial muscles has shown some promising results, especially when accompanied by physiotherapy [11, 12]. However, it remains to be clarified which muscles should be infiltrated with BTX or, in other words, which are the overactive and hypoactive muscles in PS. This point is of high clinical significance also for the physiotherapy treatments associated with BTX therapy or practiced independently as a rehabilitation strategy of its own.

In the present study, we describe the electromyographic patterns of paraspinal and axial muscles of 60 patients with classical PD and PS.

Firstly, we wanted to learn about resting state electromyographic features of different axial muscles. Furthermore, we aimed at investigating their voluntary muscle activation pattern, hypothesizing that decreased muscles' voluntary activation and recruitment pattern would reflect centrally originated unbalanced activation of axial muscles. Such results might deeply impact future therapeutic approach to PS, providing information about the best sites where to inject BTX and which muscles to strengthen during physiotherapy sessions.

## 2. Methods

### 2.1. Participants

We screened 74 in-patients with the diagnosis of probable PD based on Gelb et al. criteria [13] who met the published criteria for PS [2] and were hospitalized during the year 2014 at the Parkinson's Disease and Brain Injury Rehabilitation Department of “Moriggia-Pelascini” Hospital in Gravedona ed Uniti (Italy). All patients were on chronic antiparkinsonian therapy with dopaminergic drugs (levodopa and dopamine agonist), stable on their daily regimen over the 8 weeks prior to enrolment. All patients and their caregivers were asked to indicate the limbs side where PD motor symptoms firstly appeared and how long they have been aware of their axial bending. A neurologist expert in movement disorders evaluated all patients one hour after they took their first morning dose of antiparkinsonian medications. The Unified Parkinson Disease Rating Scale (UPDRS) [14] sections II and III were performed for all patients. Inclusion criteria were (i) probable diagnosis of PD according to Gelb et al. [13], (ii) lateral bending of the trunk (at least 10°), worsened by a prolonged sitting position or walking and completely disappearing in lying position [2], and (iii) MMSE >25. Exclusion criteria were (i) presence of clinically significant dyskinesias, (ii) neurological diseases other than PD, (iii) clinically significant psychiatric disturbances, (iv) orthopaedic spine abnormalities, and (v) present or past use of neuroleptics, lithium carbonate, dopamine receptors blocking drugs, and antidepressant or cholinesterase inhibitors.

All patients underwent spine radiogram in standing position in order to disclose the presence of orthopaedic conditions that could determine and/or worsen the lateral bending of the trunk (vertebral fractures, collapsed or wedged vertebrae, or radiological significant spine osteoporosis).

Sixty PD patients met the inclusion-exclusion criteria and were enrolled in the study.

The study design and protocol were approved by the local Scientific Committee and Institutional Review Board (Moriggia-Pelascini General Hospital, Gravedona e Uniti, Como) and were in accordance with the Code of Ethics of the World Medical Association (Declaration of Helsinki, 1967). After a complete explanation of the study protocol, a written informed consent was obtained from all patients before entering the study.

### 2.2. Electromyography

Each patient underwent electromyography (EMG) with needle electrodes (Neuroline concentric needle, 38 × 0.45 mm 1.5′′ × 26 G, Ambu, Ballerup, Denmark). Paraspinal, thoracic, and lumbar muscles (T10-L2) (PSp), rectus abdominis muscle (RA), external oblique muscle (EO), and quadratus lumborum muscle (QL) of both sides were examined. We chose these muscles since they are specifically involved in postural control and they have been widely studied in previous papers concerning PS [7, 15].

EMG was assessed with the patients lying in supine position for RA and EO muscles and on their abdomen for PSp and QL in three conditions: (a) at rest, (b) during maximal voluntary muscle contraction (MVMC) while bending in the opposite direction of the PS, and (c) during MVMC while bending in the direction of the PS. We studied patients only in a lying position to avoid the effect of body's posture and gravity on muscle activity. Before EMG evaluation, the neurologist and the physiotherapist explained to the patient how to perform the movement correctly in order to activate the specific muscles. MVMC was achieved by an encouragement of trained physiotherapists, who participated in all the exams and encouraged the patients to generate their maximal force when bending on demand.

At rest we evaluated the presence of denervation (fibrillation potentials, positive sharp waves, and fasciculations) and the presence of myopathic signs (reduced, small, or polyphasic motor unit potentials) during weak voluntary contraction. The MVMC has been provided in order to obtain information about the recruitment pattern: it was classified as normal or reduced according to, respectively, the presence of an interferential (INT) or subinterferential pattern (sub-INT). Moreover, for each muscles' pair (right and left), we defined the muscle behaviour as asymmetrical activation (AA) when a sub-INT was detected on the side opposite to the PS bending, in the presence of an INT of muscles in the direction of PS bending.

### 2.3. Statistical Analysis

Descriptive statistics are reported as mean ± standard deviation (SD) for continuous variables and as *n* (frequency percentage) for discrete variables.

Comparisons of categorical variables were carried out by the Chi-square test or Fisher's exact test when appropriate. Between groups comparisons for continuous variables were carried out by unpaired *t*-test. All statistical tests were two-tailed and statistical significance was set at *P* < 0.05. Statistical analyses were carried out using the SAS/STAT statistical package, release 9.2 (SAS Institute Inc., Cary, NC, USA).

## 3. Results

Demographic and clinical characteristics of the study group are reported in Table 1. The onset of motor symptoms was in the left limbs in 40 patients (67%) and in the right limbs in the remaining 20 patients (33%). The bending was to the right in 43 patients (72%) and was opposite to the side of motor symptoms onset in 47 out of the 60 cases (78%, *P* < 0.0001).

### 3.1. EMG Findings

The EMG did not show any spontaneous activity at rest (suggestive of acute denervation), either signs of chronic denervation and/or myopathy in all examined muscles (all EMG data are showed in Supplementary Material) (see Supplementary Material available online at http://dx.doi.org/10.1155/2015/437190).

Table 2 reports the EMG results during MVMC in the 4 muscle pairs tested when the patient was instructed to bend the spine towards and opposite to the direction of the PS, looking for INT or sub-INT.

The strongest association between bending side and AA was observed concerning EO.

Out of 17 PD patients with PS bending to the left, 10 (59%) had at MVMC an INT of the EO muscle in the direction of PS bending (*P* = 0.002) and 9 (53%) had also a sub-INT of the EO muscle opposite to PS during MVMA.

Out of 43 PD patients bending to the right, 34 (79%) had an INT of the EO muscle in the direction of PS bending (*P* = 0.0003) and 31 (72%) a sub-INT of the EO muscle opposite to PS during MVMC.

**Figure 1:**
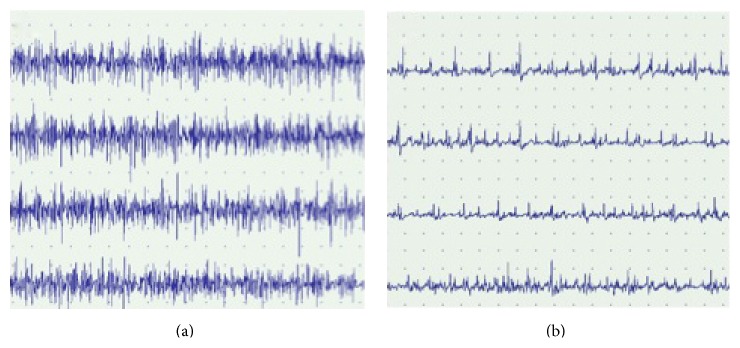
EMG of EO muscles of both sides during MVMC in a patient with PS bending to the right side. (a) Interferential pattern in the right EO muscle during MVMC. (b) Subinterferential pattern in the left EO muscle during MVMC. EMG: electromyography; EO: external oblique muscle; MVMC: maximal voluntary muscle contraction.

These data suggest that a normal function of the EO muscles ipsilateral to the PS bending is often associated to a reduced function of the contralateral EO muscle during MVMC (67% of patients had an AA) (see Figure 1 for an example).

Moreover, the classic postural attitude in PS (latero-antero flexion of the trunk with opposite axial rotation) is the movement related to the activity of EO [16].

Main findings for the EO muscle during MVMC were as follows: (i) 47 patients showed a sub-INT on the opposite to the PS bending side; (ii) among these patients, in 36 the sub-INT side corresponded to the most affected PD side; (iii) in 40 patients an AA was observed; (iv) 16 patients showed a sub-INT in the EO in the bending direction; and (v) 7 patients showed a symmetrical muscle activity.

Main findings for the RA muscle during MVMC were as follows: (i) 20 patients showed a sub-INT on the opposite to the PS bending side; (ii) among these patients, in 14 the sub-INT side corresponded to the most affected PD side; (iii) in 16 patients an AA was observed; (iv) 17 patients showed a sub-INT in the EO in the bending direction; and (v) 29 patients showed a symmetrical muscle activity.

Findings for the QL muscle during MVMC were as follows: (i) 34 patients showed a sub-INT on the opposite to the PS bending side; (ii) among these patients, in 24 the sub-INT side corresponded to the most affected PD side; (iii) in 19 patients an AA was observed; (iv) 25 patients showed a sub-INT in the EO in the bending direction; and (v) 26 patients showed a symmetrical muscle activity.

Finally, findings for the PSp during MVMC were as follows: (i) 20 patients showed a sub-INT on the opposite to the PS bending side; (ii) among these patients, in 16 the sub-INT side corresponded to the most affected PD side; (iii) in 14 patients an AA was observed; (iv) 19 patients showed a sub-INT in the EO in the bending direction; and (v) 30 patients showed a symmetrical muscle activity.

AA in EO was observed in 74% of patients aged ≤ 70 years, while it was observed in only 59% of older patients, but this difference did not reach statistical significance.

As far as the relationships with the side where motor symptoms of PD first noticed in the limbs, out of 40 PD patients more affected on the left side 28 (70%) had sub-INT of the EO left muscle and out of the remaining 20 patients affected on the right side 10 (50%) had a sub-INT of the EO right muscle (*P* = 0.039 for the comparison PD-left affected versus PD-right affected), suggesting that this relationship is much stronger in patients started their motor symptoms in the left limbs.

No significant association was observed between contralateral hypoactivation in EO muscles and PS duration, PD duration, and age.

## 4. Discussion

To the best of our knowledge, this is the largest study performed in PD patients with PS, whose postural control muscles were evaluated in supine or prone position with EMG in order to explore the relation between trunk and abdominal muscles activity at rest and during MVMC. Interestingly enough, we have not found signs of denervation or myopathy by needle EMG and as a result we see no support for peripheral contribution to the development of PS.

Moreover, we found a clear AA for the EO during the MVMC: an INT for the EO muscle ipsilateral to the direction of the PS bending and a sub-INT for the contralateral ones (40/60 patients, 67%). In contrast, no such tendency was observed for QL and RA, while 50% of patients showed a symmetrical activation of PSp during MVMC.

Previous EMG studies in patients with PS focused their attention on the paraspinal lumbar (L2–L4) and thoracic (T8–T10) muscles evaluated in standing conditions [7] and detected two different patterns of muscular activation: a hyperactivity of lumbar paraspinals ipsilateral to trunk bending side or hyperactivity of paraspinals contralateral to trunk bending side. These observations suggest that a dystonic activity could play a role in determining the bending ipsilaterally to PS and that the contralateral excessive muscle activation represents a compensatory mechanism. Tassorelli et al. found an abnormal tonic hyperactivity on the side of the trunk's deviation in abdominal oblique muscles [15], whereas Tinazzi et al. observed this phenomenon only in few patients [7]. The reason for this difference, according to the authors, might be due to the different period of onset of PS in the studied populations.

In our study, we did not find such a correlation between contralateral hypoactivation in EO muscles and PS duration.

Abnormal postures of the trunk are typical in PD, but their origins remain unclear. Trying to understand this phenomenon, we should consider the key role of the asymmetric basal ganglia functioning in the pathogenesis of PD. We found a sub-INT of EO in the side opposite to PS during MVMC and an INT of the EO of the side ipsilateral to PS.

On the basis of these observations and given the lack of signs of involvement of the peripheral nervous system, it is possible to relate the pathogenesis of PS to a “central” deficit in the recruitment of motor units connected to the most affected side as a possible consequence of the asymmetric basal ganglia outflow to the cortex.

It has been shown that the weakness, one of the signs of PD, could be related to a dysfunction in the movement programming at the level of basal ganglia [17]. Catalá and colleagues found that parkinsonian patients are not able to activate the proper muscle in order to perform the required task, suggesting a role of the basal ganglia in optimizing muscle synergy patterns [18]. These observations confirm the altered motor unit behaviour in PD: the discharge patterns of motor units are irregular and intermittent, a greater number of motor units are recruited at low thresholds, and antagonist muscles are abnormally coactivated [19].

On the basis of our data and literature evidences, it is possible to hypothesize that this dysfunctional basal ganglia-cortex-motor unit system could play a role in the pathogenesis of PS since it leads to a prevalence of the EO in the less affected side that is normally activated, with a slow development of the trunk bending in direction of this side.

Furthermore, the parkinsonian patients have a dysfunction of the somatosensory integration such as of the proprioceptive biofeedback concerning the static position [20–22], and it is interesting to note that muscle weakness itself can lead to an impairment of the proprioceptive control of human standing [23].

The impaired proprioceptive biofeedback leads to an alteration in the internal representation of verticality, contributes to the trunk deviation, and explains why parkinsonian patients with PS perceive themselves as standing up straight [21]. Sensory information has two roles: to make conscious the subject about the body's position in space and to drive the motor response [24]. We know that the joint receptors (Golgi tendon organs, free endings, Pacinian corpuscles, and Ruffini endings) monitor constantly the range of motion forces operating as proprioceptors [25], thus contributing to the perception of verticality. In PD, rigidity and bradykinesia lead to a reduction in joints' ranges of motion. This fact changes the information coming from the periphery to the cortex about the body position, impairs motor response, and participates to developing of the PS [26].

Finally, based on our EMG data, we hypothesize that the development of PS is due to a central dysfunction: the asymmetric basal ganglia outflow to the cortex leads to a reduced motor unit recruitment in the muscle of the most affected side resulting in asymmetrical muscular activity of the EO. This changes the normal posture leading to a bending position, which is not recognized by the parkinsonian patients because of a dysfunctional sensorimotor integration and that is slowly worsened by the action of gravity.

### 4.1. Rehabilitative Aspects

Based on our data, the importance of two factors on the developing process of PS is evident: the asymmetrical beginning of disease and the influence of abdominal muscles.

We argue that the prevention of PS should start at the moment of the diagnosis of PD. The patients have to start immediately a set of exercises, including stretching of PSp, in order to prevent the shortening of the muscle length and improve the strength of the same muscles. Moreover, the patients have to perform stretching exercises for the EO muscles of the less affected side, and furthermore exercises to improve the activity of EO of the most affected side could be planned. In addition, patients should perform sports activities that specifically can influence and maintain the symmetry (e.g., swimming backstroke) and avoid sports that can worsen the asymmetry (e.g., tennis). Looking at themselves in the mirror in order to correct their posture could be another simple, efficient exercise in order to increase the awareness of patients' real posture. Moreover, BTX injections in the treatment of PS should be considered, also in the early stage of the disease. In particular, after an EMG evaluation, BTX should be used infiltrating the more active EO muscle in order to reduce the asymmetry with the opposite muscles and then work with exercise to improve the muscle activity of the more affected side. We think that the PSp should never be infiltrated: our data in fact do not support a particular contribution of PSp in the pathogenesis of PS but recognize the action of these muscles in terms of compensatory activity for postural correction, suggesting that they should not be weakened.

### 4.2. Study Limitations

The most important limitation in this study is the lack of an experimental group treated with a specific rehabilitation approach. In this study we suggest only a possible protocol that will be tested in further studies. We did not evaluate patients in standing position since other studies that were conducted with this approach did not reach conclusive results. Our aim was to increase the knowledge about PS pathogenesis and we had limited our attention to muscle activity in lying or prone position. In further studies it will be interesting to evaluate and compare the different aspects of muscle activity in standing or even walking to lying position.

## 5. Conclusion

We hypothesized that PS might be due to a central dysfunction (decreased muscle activation-weakness) which causes dissynchronisation of axial muscles activity. Our results support this hypothesis, demonstrating that inability to generate maximal force of the EO muscle in the most affected parkinsonian side of the body is the primary cause of central dissynchronisation which leads to the bending of the spine while sitting, standing, and walking. These results have significant implication to physiotherapy and the use of BTX in the treatment of PS. The aim should be to strengthen the weaker muscle by physiotherapy or to weak the stronger muscle by BTX injection. Future interventional studies should deal with this proposal for treatment.

## Supplementary Material

EMG data during MVMC.

## Figures and Tables

**Table 1:** Clinical and demographic data for the study population (*n* = 60).

Variable	Mean ± SD
Age	68.1 ± 7.1
Hoehn-Yahr stage	2.6 ± 0.5
Disease duration	9.7 ± 4.4
Onset of PS	8.1 ± 6.6
UPDRS II	15.6 ± 5.1
UPDRS III	21.9 ± 5.3
Daily levodopa equivalent dose (mg)	679.5 ± 322.6

PS: Pisa Syndrome; UPDRS: Unified Parkinson's Disease Rating Scale.

**Table 2:** Summary of muscle activation data during MVMC.

	Subinterferential pattern opposite to bending side	Subinterferential pattern in the direction of bending side	Asymmetrical activation
EO	47 (78%)	16 (27%)	40 (67%)
RA	20 (33%)	17 (28%)	16 (27%)
QL	34 (58%)	25 (43%)	19 (32%)
PSp	20 (33%)	19 (33%)	14 (23%)

MVMC: maximal voluntary muscle contraction; EO: external oblique muscle; RA: rectus abdominis muscle; QL: quadratus lumborum muscle; PSp: paraspinal muscles.
