# Supplementary material for: Pisa Syndrome in Parkinson's Disease: Electromyographic Aspects and Implications for Rehabilitation
Source: Parkinsons Dis. 2015 Nov 23;2015:437190. doi: 10.1155/2015/437190 (PMC4670865; doi:10.1155/2015/437190)
Supplement: Supplementary file 1 — EMG data during MVMC. [file 437190.f1.pdf]

Table 2. EMG data during MVMC (0: sub-interferential pattern; 1: Interferential pattern).

ID	EO left	EO right	QL left	QL right	RA left	RA right	PSp left	PSp Right	Most affected side of PD symptoms	PS direction
1	0	1	0	1	0	1	1	1	R	R
2	0	1	1	0	0	1	1	0	R	L
3	0	1	1	0	0	0	0	1	L	R
4	1	0	1	1	1	0	1	0	L	R
5	0	1	0	1	1	0	1	0	L	R
6	1	1	1	0	1	0	1	1	L	L
7	1	0	0	1	0	1	0	1	L	R
8	0	1	0	1	0	1	0	1	L	R
9	0	1	0	1	0	1	1	0	L	L
10	0	0	1	0	1	0	1	1	R	L
11	1	1	0	0	0	1	1	0	L	R
12	0	1	1	0	1	1	1	0	L	R
13	1	0	0	0	0	0	0	1	L	L
14	0	1	1	1	0	1	1	1	L	R
15	1	0	0	1	1	1	0	1	R	L
16	0	0	0	1	1	0	0	1	L	R
17	0	1	1	0	0	1	1	1	R	L
18	0	1	0	0	1	1	0	0	L	R
19	0	1	1	1	1	0	0	1	L	R
20	0	1	0	1	1	1	0	1	L	R
21	0	1	0	0	1	1	1	0	L	R
22	0	1	1	0	1	1	1	0	L	R
23	0	1	1	1	1	1	1	0	L	R
24	0	1	0	0	1	1	0	0	L	R
25	0	1	0	1	1	1	1	1	L	R
26	0	1	0	0	0	0	1	1	L	R
27	1	1	1	0	1	0	1	0	L	R
28	0	1	0	1	1	1	1	1	L	R

29	0	1	0	0	1	1	1	0	R	L
30	1	0	1	1	0	1	1	1	R	L
31	0	1	1	1	1	1	1	1	L	R
32	1	0	1	0	0	1	1	1	L	L
33	0	0	0	1	0	1	0	1	R	R
37	0	1	0	NA	1	1	1	NA	L	R
35	0	0	NA	NA	0	1	0	1	L	R
36	1	0	1	1	0	1	1	0	R	L
37	1	0	1	1	1	1	1	1	L	L
38	0	1	0	1	0	1	1	1	R	R
39	0	1	0	1	1	1	0	1	L	R
40	0	0	0	0	1	0	0	1	R	L
41	1	0	1	1	1	0	1	1	R	L
42	0	0	0	0	0	1	0	0	L	R
43	0	1	0	1	0	0	1	1	R	R
44	0	1	0	0	1	1	0	1	R	R
45	1	0	1	0	1	0	1	0	L	R
46	0	1	0	0	1	1	1	1	L	R
47	1	0	1	1	1	1	0	1	R	L
48	1	0	0	1	1	0	0	1	L	R
49	0	1	0	1	1	1	1	1	R	R
50	0	1	1	0	1	1	1	1	R	R
51	1	0	1	0	1	1	1	1	L	R
52	1	0	1	1	1	1	1	1	R	L
53	0	0	0	0	1	0	0	0	R	L
54	0	1	1	0	1	1	1	1	L	R
55	0	1	1	1	1	1	1	1	L	R
56	0	1	1	0	1	1	1	1	L	R
57	0	1	1	1	0	1	1	1	L	R
58	1	1	1	1	0	1	1	1	L	R
59	0	1	0	0	1	1	0	0	R	R
60	0	1	0	0	1	1	1	0	L	R

Abbreviation: *MVMC (maximal voluntary muscle contraction); R (right); L (Left); NA (Not assessed); EO (external oblique muscle); QL (quadratus lumborum muscle); RA (rectus abdominis muscle); PSp (paraspinal muscles); PD (Parkinson's disease); PS (Pisa Syndrome).*
